# Births and deaths in Sidama in southern Ethiopia: findings from the 2018 Dale-Wonsho Health and Demographic Surveillance System (HDSS)

**DOI:** 10.1080/16549716.2020.1833511

**Published:** 2020-10-29

**Authors:** Hiwot Abera Areru, Mesay Hailu Dangisso, Bernt Lindtjørn

**Affiliations:** a School of Public Health, Hawassa University, Hawassa, Ethiopia; b Centre for International Health, University of Bergen, Bergen, Norway

**Keywords:** Demographic transition, fertility, age reliability index, qualitative, southern Ethiopia

## Abstract

**Background:**

Sidama is one of the most densely populated areas in Ethiopia. Information about the demographic characteristics is scarce, and most studies were census based on interviews. Earlier population studies from Ethiopia did not sufficiently address the validity of measuring births, deaths, and age-composition.

**Objective:**

To investigate the population characteristics in Sidama with an emphasis on fertility estimates, age, and death reporting.

**Methods:**

This is a mixed-method cross-sectional study, conducted in Sidama in southern Ethiopia, using baseline data of newly established Dale-Wonsho Health and Demographic Surveillance System site in 2018. We used quantitative data of 5179 randomly selected households having 25,144 individuals. We collected information on deaths in the same study period and population from the traditional burial associations (*Iddir*). Qualitative data were collected using focus group discussions, and in-depth interviews. Life tables, age reliability indices and logistic regression were used to analyse the data.

**Results:**

The total fertility rate was 2.9 children/woman, the crude birth rate was 22.8/1000 population and the crude death rate was 5.2/1000 population. The dependency ratio was 66/100 working-age population. Urban residents had higher birth rates (OR = 1.4 (95% CL: 1.05–1.78), and women with basic education had lower birth rates (OR = 0.6 (95% CL: 0.46–0.78) compared to those with no education. The age accuracy indices showed unreliable age reporting. The number of deaths increased from 29 to 132 when death reports from the *Iddirs* were included. There was under-reporting of neonatal and deaths of young children. Substituting national and regional mortality estimates, the life expectancy declined to an average of 53 years (range 48–58 years).

**Conclusion:**

The fertility rate in Sidama is lower than previously reported and is affected by age, residence and education. As we have identified important measurement and reporting errors, future demographic surveillance sites should consider these limitations.

## Background

Although fertility started to decline in Asia and Latin America about 60years ago, it has only started to fall in Africa over the past decade. Thus, an increase in the projected population of Africa is still high, mainly because of the high levels of fertility [1].

Demographic transition theory describes a series of stages that a population experiences due to changes in birth and death rates. During these stages, the population growth and demographic structure shift from a high to a low state of mortality and fertility. Most often, the decline in mortality precedes the decline of fertility [2].

Ethiopia, has during the past decades, experienced high economic growth [3,4] and is undergoing a demographic transition from high to low mortality and fertility since the 1990s. The population growth rate is expected to decline further, and life expectancy is expected to increase. The reductions in mortality and fertility rates are more rapid in Ethiopia than in other sub-Saharan African countries [3–5]. This also creates a window of opportunity for the country by increasing the proportion of the working-age group population to accelerate economic growth [6].

In addition to population size, fertility, mortality and migration, age and sex structure can also influence the number of births, deaths, and moves of the population taking place [2]. Under such a view, reduced fertility can be viewed as an important factor contributing to economic development [7–9]. Some even argue that reduced population growth is a necessary condition for achieving the development aims, such as the Sustainable Development Goals 1–4 [10,11]. However, other scholars believe that fertility reduction is a consequence, rather than a cause, of other underlying changes, such as wealth and literacy, that produce development [12,13]. Births, deaths, and migration are considered the determinants of population change. Change in one or more of these components will result in a change in the population size or structure [2]. Countries generate information from census, vital events registration, population-based surveys, and surveillance systems. This is crucial for evidence-based policy formulation and decision, program planning, and practice. Most developed nations have regular and well-established registration systems as sources of information [14]. Unfortunately, in many developing countries, there is a deficiency in national civil registration and vital statistics system. Ethiopia launched a vital event registration system in August 2016, yet the coverage of birth registration in southern Ethiopia is only 3% [15–17]. Therefore, such countries still depend on data from health facility reports, census and demographic and health surveys for planning and allocation of resources [15,18–22].

Health and Demographic Surveillance Systems (HDSSs) can provide valuable data for long term follow-up of the specifically defined population [23–25]. To get an accurate measure of fertility and mortality, eliminating the effect of age structure and population size is essential. Distortion in age distribution may be caused by past changes in the level of mortality, fertility, and migration or it may be due to errors. The error may also be due to omission or misreporting of individuals’ age. This is a common problem in developing countries [2].

In Ethiopia, the crude birth rate has fallen from 48.3 to 32.3 per 1000 population from 1985 till 2015, and the total fertility rate is now 4.3 children per woman [26]. The crude death rate also has fallen from 19.0 per 1000 population in 1985, to 6.7 per 1000 population in 2015. The infant mortality rate has decreased by 70%, and the under-five mortality decreased by 74% since 1985 [26]. However, neonatal mortality decreased by 52% [16,27]. Correspondingly, the median life expectancy has risen to 66 years in 2015, from 46 years in 1985 [26]. Various studies done in Ethiopia have shown that women who are more educated, having better income and living in urban areas, have lower fertility than their counterparts [16,28].

Even though there were different health and demographic surveillance conducted in Ethiopia, the findings are inconsistent across the country. The study currently conducted in Sidama in southern Ethiopia focus on mortality and fertility, where no demographic studies had been done before. A study done in Butajira rural health project in Ethiopia showed the acceptable level of error for measuring births, deaths and age. However, they failed to show the errors encountered in the baseline population because of measurement or program errors and considered them as limitations [29]. We believe that addressing such potential measurement errors in data collection in demographic studies is crucial to avoid inaccuracies in subsequent data collections on the same populations. It also helps to get estimates that reflect the real population dynamics [30,31].

This study aims to investigate the population characteristics in Sidama with an emphasis on fertility estimates, age, and death reporting.

## Methods

### Study area

This study was done in two districts in Sidama Region, one of the most densely populated areas of southern Ethiopia with 533 persons/km^2^ [32,33]. The Region accounts for 4.0% of the National population [34]. Ninety-five per cent of the population speaks the native Sidama language. More than 84% of the population is protestant in their religion, and the traditional religion followers constitute almost 3% of the total population [35]. The Dale district (woreda) covers an area of 30,212 km^2^, with a population of 268,839 people and an estimated 53,768 households. It has 36 rural and 2 urban kebeles (the lowest administrative structures). Wonsho district covers 14,528 km^2,^ and 129,730 people live in 17 rural and one urban kebele in 21,857 households. Even if agriculture is a prominent economic sector of the region, farming is traditional [35,36] Both districts are known for their coffee and crop production. Dale district has 10 health centres and 33 health posts, while Wonsho district has 5 health centres and 17 health posts [35,36].

### Dale and Wonsho Health and Demographic and Surveillance site

There has been an increase in the number of HDSSs in Ethiopia [20]. And in 2017, Hawassa University established its own Health and Demographic Surveillance System site in Sidama in Dale and Wonsho districts. This site aims to fill evidence gaps by generating community-based health data in southern Ethiopia. The D-W HDSS also signed a memorandum of understanding with the Ethiopian Public Health Institute (EPHI) and is considered as one of the surveillance sites which generate longitudinal health and demographic data in Ethiopia.

Ten of the kebeles were selected by simple random sampling technique from Dale and Wonsho districts, which were incorporated based on their agricultural practice and environmental characteristics. The urban kebeles were selected purposively; one from each district to incorporate population from towns. The estimated households in the sample are around 12,500, with a population of 60,000 people. The surveillance site is called Dale-Wonsho Health and Demographic Surveillance Site (D-W HDSS). (The map of the study area is attached as supplementary material 3).

### Study design

This study used a mixed-method design, using both quantitative and qualitative data. A cross-sectional study design was employed to identify the population characteristics. A qualitative study was undertaken to explore perceptions regarding age reporting, during vital events such as births, deaths, and to triangulate these findings with the quantitative findings. Triangulation is the planned use of two or more methods independently, in investigations of the same event, to strengthen the validity of the results. It helps in mitigating the biases and limitations faced by using a single method [37]. It was done through focused group discussions (FGD) and in-depth interviews (IDI) on variables that required a comprehensive understanding of the community about reporting of events.

### Sample size and sampling procedure for quantitative data

In this study, we used a subsample of the D-W HDSS census data. The D-W HDSS policy allowed us to use about 40% of their data for this study. We checked for sample adequacy by taking the national crude death rate estimate of 6.7 per 1000 population, 95% confidence interval, power of 80%, and unexposed/exposed ration of 1. The sample size required using these assumptions was 14,600; the margin of error for this sample was 0.13%. The sample size used for the current study was 25,144 individuals. We initially took a weighted sample from each kebele based on their population size. Then simple random sampling using a random number generator was used to select the specific house numbers from each kebele. For this study, we included 41% of randomly selected households from the D-W HDSS baseline census, and we used the collected data of the variables we wanted to analyse. These processes made our sample representative. (Refer supplementary material 2 for detailed sampling procedure).

The source population for this study were all households in Dale and Wonsho districts, and the study population were the households in the twelve randomly selected kebeles of D-W HDSS.

### Study unit

The households were selected by a random number generator and included 5,179 households constituting 41% of the total households (Table 6 is attached as a supplementary material 2).

### Sampling procedure for the qualitative study

Purposive sampling was used to select participants (community leaders, religious leaders, experts) for the in-depth interview (IDI) and Focus Group Discussions (FGD) to explore the communities’ perception and experience on mortality, birth and age reporting. This method was used to supplement and strengthen the quantitative findings of this paper. A total of 8 IDIs, four from each district, were conducted. A total of 48 individuals, who came from each of the 12 kebeles participated in four different FGDs. Two of the FGDs were for males and the other two for females, having both younger and older age categories to address the issues across the different segment of the population.

### Data collection procedure

Data were extracted from the Dale-Wonsho Health and Demographic Surveillance site (D-W HDSS) database in Hawassa University. The database consists of six tables which contain background characteristics, birth information, death information, maternal information (FP and ANC), disease information and household characteristics. For this study, we used all except maternal and disease information tables.

A de-facto census for a population who actually is present during the enumeration [2], was done in 2017 from the selected 12 HDSS kebeles over 6 month period. There were two local data collector and one supervisor assigned for house visits in each kebele to collect the data. The baseline information consisted of background characteristics, information on births, deaths, diseases, and household characteristics. Data on migration were not collected at baseline. Then data were entered into HRS-2 software [38] by four trained data clerks. For this study, 41% of the households were selected and exported to Stata version 13 for cleaning and analysis.

Mortality data on the same population and for the same period (deaths occurring within 12 months before the HDSS survey) was collected from ‘Iddirs’ of all kebeles. *Iddirs* are traditional voluntary organizations created with a primary purpose of offering mutual aid in burial issues, even though they may be involved in dealing with other community matters [39]. The *Iddirs*’ death records were used to find out missed mortality cases from HDSS and to cross-check the accuracy of what was reported. However, the *Iddirs* had only the names, sex and dates of deaths of the deceased individuals. After having obtained the addresses of the households, the data collectors went to the deceased family’s household to confirm and get additional information on the age of the deceased, and the possible cause of death.

**Operational definitions** are attached as supplementary material 1 [60−63].

### Data analysis

The analysis was done using Stata/IC version 13.0 software (StataCorp LP., College Station, Texas, USA) and Microsoft excel. Descriptive analysis was done on variables describing the population characteristics. The population pyramid was constructed to see the age and sex composition of the population. Age reliability was calculated using Whipple’s index for ages ending by zero and five, Myer’s blended index for all ages ending from zero to nine, and United Nations Joint Score for five years age groups up to age 70 years for both sexes [2,40,41]. Principal component analysis (PCA) was done to establish a wealth index. Eighteen variables were dichotomized and considered for PCA. The variables were ownership of radio, television, refrigerator, telephone, bicycle, motorcycle, car, cattle, carriage animals, sheep/goats, chickens and land; and material of the roof and the floor, source of light, source of drinking water, toilet facilities and cooking material. Sensitivity analysis for children’s mortality and crude birth rates was done by substituting estimates from regional and national studies, to crosscheck the consistency of the results. For mortality, the infant mortality rate (IMR) of 43 deaths/1000 live births and child mortality rate of 12 deaths/1000 live births from the national estimate were used. Besides, the southern Ethiopia’s IMR of 65 deaths/1000 live births and 25 deaths/1000 live births were used to create different scenarios [16,42]. To calculate the life expectancy for our study, we used southern Ethiopia’s numbers of IMR of 65 deaths/1000 live births with corresponding 42 infant deaths and child mortality of 25 deaths/1000 live births with corresponding 39 child deaths [16]. Similarly, the national IMR of 43 deaths/1000 live births with corresponding 28 infant deaths and child mortality of 12 deaths/1000 live births with corresponding 19 deaths were substituted to calculate different life expectancy scenarios [42]. For births, the national crude birth rate (CBR) of 31.8 births/1000 population and Dabat HDSS’s CBR of 28 births/1000 population were used for comparison [16,43]. Spectrum, a modelling and planning software for improved health, version 5.761 software (Avenir Health, Glastonbury, CT, USA) was used to estimate the projected total fertility rate by comparing our data (2.9 births/woman) with the national numbers that are used in the software [44]. Bivariate and multivariable logistic regression was done to assess factors associated with fertility.

For qualitative data, the recorded audio in *Sidamu Afoo* was translated to Amharic by fluent speakers of both languages. Then the Amharic version was translated and transcribed into English. We used ATLAS. ti 6.2 qualitative analysis software (ATLAS. ti, GmbH, Berlin) to code and analyse the data by two of the researchers. Several emerging themes were generated, such as ways of age estimation, culture on death reporting and roles in a community.

### Data management and quality control

The data clerks were trained about the protocol and how to extract the data from the central database. The data were initially cleaned manually by the principal investigator. The consistency was checked for the completeness of the variables and mismatch between the questionnaire and the data entered in the HRS-2 for the selected households. When inconsistencies were found, the PI went to the study site and asked the data collectors for clarification. For incomplete data like mortality, data was recollected by visiting the local community’s organizations performing burial services called ‘*Iddir*’ from all the kebeles. The PI also assured the quality of the data on 10% of randomly selected deaths reports by going back to the selected households.

For qualitative data, after reviewing the literature, an interview guide was prepared in consultation with an experienced researcher who knew the Sidama culture and lived in the locality. Then, it was formatted in English and translated to the local language (*Sidamu Afoo*) and back-translated to English to check its consistency. One experienced interviewer having a BSc degree in public health and an assistant, who spoke the local language fluently were trained by the principal investigator on the contents of the interviews. Moreover, the assistant was trained in recording and taking field notes. A tape recorder was used to capture all information from the participants, and this was later translated into English.

## Results

### Description of background characteristics

The total number of households in this study was 5179, and the number of individuals living in these households was 25,144 people. Six out of ten people were below the age of 25 years (15,542 people), and half of the population were not married (10,063 people). Most of the population, 96.5% (24,258 people) were Sidama by ethnicity, 89% (22,322 people) were Protestants by religion and 83% (20,817 people) lived in rural areas. From those eligible for educational assessment, 35% (7,596 people) had attended grade 1–4 (primary cycle), and only 2.0% (492) had attended higher education. (Table 7 is attached as supplementary material 3).

### Demographic descriptions

Table 1 describes some of the demographic indicators of the population. The average number of individuals in a household was 4.9. The total fertility rate was 2.9 children per woman. The crude death rate of the population was 5.2 deaths per 1000 population. The dependency ratio of the population was 66 dependents per 100 working-age population.

**Figure 1.**
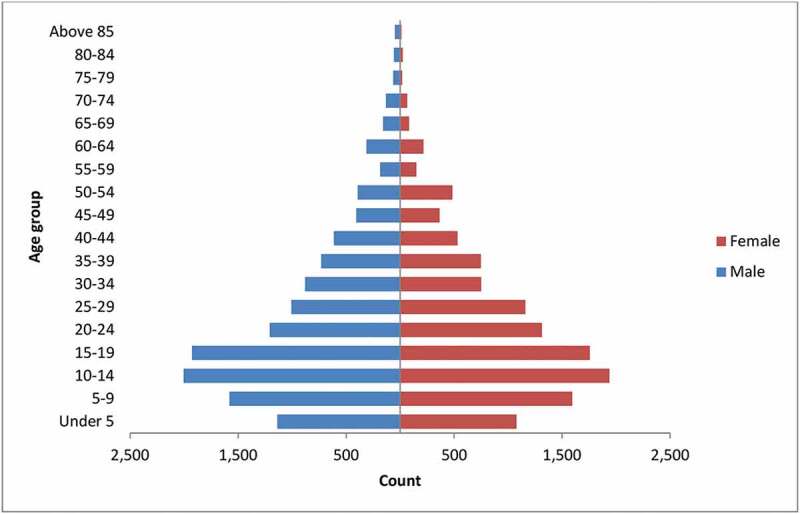
Population pyramid of Dale and Wonsho districts 2017/18

**Table 1.** Some demographic indicators of Dale and Wonsho districts, 2017/18, Sidama, Ethiopia

Indicators	Calculations	Value
Total number of live births in a year	572 live births/573 births	99.8%
Crude Birth Rate (CBR)	573 annual birth/25,144 mid-year population	22.8 births per 1000 population
Total Fertility Rate (TFR)	TFR = 5*ASFR/1000	2.9 child per woman
General fertility rate (GFR)	573 births/6,628 reproductive age women	86.5 per 1000 live birth
Gross Reproductive Rate (GRR)	GRR = 5*Female ASFR/1000	1.33 daughters per woman
Net Reproduction Rate (NRR)	GRR*Probability of surviving to the mean of age specific fertility distribution(30.06 years in our case) 1.33*0.964	1.28 daughters per woman
Crude Death Rate (CDR)	132 annual deaths/25,144 mid-year population	5.2 per 1000 population
Infant Mortality Rate	7 deaths of infants/573 live births in a year	12.2 per 1000 live birth
Child Mortality Rate 1–4	3 deaths of children aged 1–4 years/573 live births in a year	5.2 per 1000 live birth
Under five morality rate	10 deaths of under five years children/573 live births in a year	17.5 per 1000 live birth
Crude rate of natural increase	22.8 deaths-5.2 deaths	1.8 per 100 population
Median age		18 years
Dependency ratio	9,335 under 15 years + 664 elderly/ 15,145 working-age population	0.660 (66.0/100 working population)
Young dependency ratio	9,335 under 15 years/15,145 working-age population	0.616 (61.6/100 working population)
Old dependency ratio	664 elderly/15,145 working-age population	0.044 (4.4/100 working population)
Women of reproductive age group	Women aged 15–49 years of age	6,628 (26.4%)
Child/woman ratio	2217 under five year children/6628 reproductive age women	0.334 (334 per 1000)
Sex ratio (birth)	305 male infants/268 female infants	114
Sex ratio (total)	12,848 male population/ 12,296 female population	104
Life expectancy at birth (both sexes)	The detail is annexed as a supplementary file 7, Table 10	62 years
Life expectancy at birth (Male)	The detail is annexed as a supplementary file 7, Table 11	63 years
Life expectancy at birth (Female)	The detail is annexed as a supplementary file 7, Table 12	60 years
Persons per households	25,144 individuals/5179 houses	4.9

The population pyramid was typical of developing countries with a wider base and narrow tip (Figure 1).

### Age preference of Dale and Wonsho districts’ population

The Whipple’s index for age with the terminal digit of five was 274 and for terminal digit zero was 323. The Myers’ blended index for each age ending from zero to nine was 32. Similarly, the United Nations Age-sex Accuracy Index (UNAI) or Joint Score (UNJS) was 110. These indices showed that the age estimation as being unreliable, falling into the very rough category. (Please see the supplementary material 1 for detail information of the indices).

### Result from qualitative study on age and death reporting

The common themes that emerged from FGDs and IDS were: the traditional way of age estimation, annual holidays, historical events, birth certificate, infant death, death of elderly, taboo, social security, full human being, mourning, reducing age, exaggerating age, respect, approval and role in a community.

In Sidama, age determination was estimated by considering historical and cultural events occurring in the communities. The major traditional way of estimating age was using the age-generation ‘Luwa’, which is a system of local leadership changed in a fixed interval. The Sidama new year festival, ‘Fichee Chambalala’, regime changes, and wars were important landmarks for calculating the age of a person. These all gave the approximate age of the person. However, currently, because many people were getting educated, they have certificates documenting their children’s birth date. Only nine out of 56 respondents said the community uses birth certificate for age estimation.

Death of the infants was usually considered a taboo in the community. There was a belief not to consider infants as ‘a full human being’, or ‘a mature person’ and mourning their deaths might bring another bad event for the family. This means deaths of infants might not have been recorded.

The age reporting among males and females had also some differences, mainly due to cultural factors. Young women tended to reduce their age, especially when they are yet to get married or expected to bear many children and get approval by the in-laws. However, males tend to exaggerate their age, especially at young and old age due to security and social respect obtained.

### Description of births

Of the 573 births, 563 (98.3%) were singleton deliveries, 572 (99.8%) were live births, and 248 (43.3%) were deliveries outside health institutions (Table 8 is attached as supplementary material 4).

### Description of deaths

There were 58 deaths recorded in the census of 12 kebeles from the HDSS database of which 29 were in our subsample. However, when we collected the data from the ‘*Iddirs*’, we found a total of 265 deaths (78% of the deaths were not found in the database), of which 132 were in our sub-sample. Of the132 deaths, 66.7% (88 deaths) occurred outside health institutions. There were 10 deaths (7.6%) among children under the age of five years. From the oral report of the participants, for 22.7% (30 deaths) the cause of death is unknown (Table 2). (See the causes of deaths in supplementary material 5).

**Table 2.** Death characteristics of Dale and Wonsho districts’ population, 2017/18, Sidama, Ethiopia (N = 132)

		Male	Female
Variables		N (%)	N (%)
Deaths in the last one year		74 (56.1)	58 (43.9)
Place of death			
	Health institution	28 (63.6)	16 (36.4)
	Home	41 (50.6)	40 (49.4)
	On the road	3 (75.0)	1 (25.0)
	Others	2 (66.7)	1 (33.3)
Age at death			
	0–4	4 (5.4)	6 (10.3)
	5–14	3 (4.1)	1 (1.7)
	15–24	2 (2.7)	2 (3.4)
	25–34	4 (5.4)	3 (5.2)
	35–44	6 (8.1)	7 (12.1)
	45–54	7 (9.5)	9 (15.5)
	55–64	10 (13.5)	6 (10.3)
	65–74	9 (12.2)	10 (17.2)
	75–84	11 (14.9)	9 (15.5)
	≥85	18 (24.3)	5 (8.6)

Because we registered under-reporting of deaths in the census, we did a sensitivity analysis to evaluate age-specific mortality and crude birth rate. Using information from earlier national and regional estimates, we estimated the under-reporting of infant and child deaths and the number of live births in our study (Table 3).

**Table 3.** Sensitivity analysis of birth and death estimates using different age specific mortality rates and crude birth rates, 2017/18, Sidama, Ethiopia

		Rates used for comparison		
Age group	Assumptions	Age specific mortality rates	Number of deaths	Difference from the current cases (possible number of unreported deaths)
Infant (<1 years)	Current study	11	7	
	EDHS national	43	28	21
	EDHS regional (Southern Nations, Nationalities and Peoples Region)	65	42	35
Children (1–4)	Current study	2	3	
	EDHS national	12	19	16
	EDHS regional (Southern Nations, Nationalities and Peoples Region)	25	39	36
All population	Current study	Crude birth rate	Number of births	
		22.8	573	
	EDHS national	31.8	700	127
	Dabat HDSS	28	704	131

Table 4 shows that the life expectancy at birth reduced from 62 years to an average of 53 years (range 48 to 58 years) when substituting previous national and regional estimates of infant and child mortalities. (The life table information is presented in supplementary material 7).

**Table 4.** Sensitivity analysis of life expectancy at birth by substituting different mortality estimates, 2017/18, Sidama, Ethiopia

x	n	P_x_	D_x_	e_x_
**Scenario 1: Using infant and child mortality of current study**				
0	1	641	7	61.79769
1	4	1576	3	61.96097
**Scenario 2: Using infant and child mortality of EDHS, SNNPR 2016 estimates**				
0	1	641	42	48.04314
1	4	1576	39	53.0278
**Scenario 3: Using national infant and child mortality of 2019 mini EDHS estimates**				
0	1	641	28	54.63236
1	4	1576	19	57.99067

Where; X = exact age; n = interval between two exact ages stated in years; Px = Population in x, x + n age group; Dx = number of deaths in x, x + n age group; e_x=_Average Number of years a person aged x has to live.

### Determinants of fertility

Table 5 showed that age, educational status, religion, and area of residence were associated with fertility. The odds of women aged 25–29 giving birth were 6.2 (4.37–8.72) times higher than the odds of women aged 15–19 giving birth. Women who had attended basic education had lower odds of giving birth by 40% (OR = 0.6; 95% CI: 0.46–0.78) when compared to those who were uneducated. The odds of urban residents giving birth were 1.4 (1.05–1.78) higher than the odds of rural residents giving birth.

**Table 5.** Crude and Adjusted Odds Ratio of the associations between characteristics of reproductive age group women and fertility in Dale and Wonsho districts, 2017/18, Sidama, Ethiopia (N = 6628)

	Gave birth last year				
	Yes	No			
Variables	N (%)	N (%)	COR (95% CI)	AOR (95% CI)	P-value
**Age Group**					
15–19	44 (7.7)	1713 (28.3)	Ref	Ref	
20–24	167 (29.1)	147 (18.9)	5.67 (4.03–7.97)	5.41 (3.84–7.62)	<0.001
25–29	166 (29.0)	994 (16.4)	6.50 (4.62–9.15)	6.18 (4.37–8.72)*	<0.001
30–34	97 (16.9)	654 (10.8)	5.77 (4.00–8.34)	5.54 (3.80–8.08)*	<0.001
35–39	72 (12.6)	676 (11.2)	4.15 (2.82–6.10)	3.76 (2.52–5.61)*	<0.001
40–44	24 (4.2)	508 (8.4)	1.84 (1.11–3.05)	1.67 (0.99–2.82)	0.05
45–49	3 (0.5)	363 (6.0)	0.32 (0.10–1.04)	0.28 (0.08–0.91)*	0.03
**Ethnicity**					
Sidama	555 (96.9)	5818 (96.1)	Ref.	Ref.	
Non Sidama	18 (3.1)	237 (3.9)	0.80 (0.49–1.30)	0.77 (0.42–1.41)	0.41
**Religion**					
Protestant	522 (91.1)	5367 (88.6)	Ref.	Ref.	
Orthodox	12 (2.1)	223 (3.7)	0.55 (0.31–1.00)	0.47 (0.23–0.96)*	0.04
Muslim	6 (1.0)	143 (2.4)	1.02 (0.69–1.51)	1.14 (0.75–1.71)	0.54
Others	29 (5.1)	293 (4.8)	0.60 (0.31–1.14)	0.61 (0.32–1.18)	0.14
**Educational Status**					
Illiterate & read-write	191(33.3)	1806 (29.8)	Ref.	Ref.	
Basic education	107 (18.7)	1608 (26.6)	0.63 (0.49–0.81)	0.60 (0.46–0.78)*	<0.001
Primary cycle	170 (29.7)	1726 (28.5)	0.93 (0.75–1.16)	0.85 (0.67–1.09)	0.21
Secondary cycle	79 (13.8)	778 (12.8)	0.96 (0.73–1.26)	0.84 (0.61–1.16)	0.29
Higher Education	26 (4.5)	137 (2.3)	1.79 (1.15–2.80)	1.18 (0.72–1.93)	0.51
**Wealth Index**					
Lower quantile	166 (29.0)	2033 (33.6)	Ref.	Ref.	
Middle quantile	187 (32.3)	2019 (33.3)	1.13 (0.91–1.41)	1.16 (0.92–1.45)	0.20
Upper quantile	220 (37.0)	2003 (33.1)	1.35 (1.09–1.66)	1.23 (0.94–1.60)	0.13
**Residence**					
Rural	436 (8.1)	4970 (91.9)	Ref.	Ref.	
Urban	137 (11.2)	1085 (88.8)	1.44 (1.18–1.76)	1.37 (1.05–1.78)*	0.02

* Significant association

## Discussion

Our study shows that fertility in Sidama is lower than in previous studies. Low fertility was associated with individual factors of the women such as age, educational status and area of residence. This study also revealed that there is inaccuracy in age and death reporting.

One of the strengths of this study is using a standard INDEPTH Network approved tools [45], and using a random sampling of the population in Sidama. Secondly, this study included both mortality and fertility with major demographic indicators. Thirdly, we used both qualitative and quantitative data collection methods, which allow us to triangulate and identify the information gaps in the data set.

The first limitation of this study was using respondents own word for age reporting. Age was estimated based on a memory of different events than recordings, therefore, resulting in unreliable age indices. We believe there was a severe under-reporting of deaths of infants, children and stillbirths. Sometimes it is difficult to distinguish between stillbirths and neonatal deaths. And as it is seen from the sensitivity analysis, if there were hidden stillbirths and neonatal deaths, the number of births could be higher than recorded. Therefore, the fertility rates could be higher than we report. We supplemented the DHS data by collecting data from burial groups and by doing a qualitative study as a form of verifying and validating the data. Furthermore, there was no information collected on migration, so we could not calculate the population change due to population movements. From other areas of southern Ethiopia, we know that migrations can be seasonal and large [46,47].

Around one-fifth of the population in this study didn’t attend formal education. However, in studies done in Gilgel gibe HDSS in Ethiopia, and Ghana, an uneducated population constituted more than half of the population [18,48]. This difference could be ascribed to the time gaps between the studies and the governments’ strategy to expand educational coverage in the last decades.

In this study, the total dependency ratio was lower than the 2015 national estimate and the 2012 Kersa HDSS [26,49]. This might be due to the regional variations in fertility and the misreporting of age on lower and higher age group in our study site. It also might be an indication of fertility transition where there is a decline in fertility, which leads to an increase in the working-age group.

The crude birth rate (CBR) in this study was lower than the findings of EDHS, and other HDSS sites in Ethiopia [16,43,48,50]. This difference might be explained by the use of three years of data preceding the survey in EDHS and the use of censuses in other HDSS sites. Moreover, this study used twelve months of data preceding the census. Besides, our study population is more rural than urban, so it’s expected to have higher CBR than urban HDSSs [51]. The cultural issues related to probable severe under-reporting of stillbirths and neonatal deaths probably explain a part of our low CBR.

The Total Fertility Rate (TFR) in Sidama was 4.1 children per woman in the 2007 national census, which is lower than the 2016 National Demographic and Health Survey report [16,52]. Yet, the total fertility rate in our study was 2.9, which is lower than both estimates. This might suggest that there might be a lower fertility trend in the area. The structure of the population pyramid may also suggest declining fertility rates.

The crude death rate (CDR) in this study was lower than the result of other HDSS sites in Ethiopia and Africa. The CDR ranged from 6.1 to 8.0 per 1000 population in other HDSS sites [43,48,50]. It might be due to the under-reporting of neonatal and children deaths in the study area. Moreover, the deaths missed by both the HDSS data and *Iddirs* records might also lower the CDR in the area.

There was discrepancy between the number of deaths collected by the D-W HDSS and collected from *Iddirs*. The reason for this difference might be due to the data collection methods implemented. D-W HDSS used face to face interviews, while our study went to the traditional institution where deaths are recorded. In a community where death reporting is associated with a taboo, the number of death reported by the family of the deceased might yield a lower number.

In this study, the rate of crude natural increase was below 2 per 100 populations which is lower than other HDSS sites in Ethiopia and Africa [48,50,53]. In addition to an earlier explained under-reporting of births, the difference between these results might be due to the lower crude birth rate attributed to increased contraceptive utilization over the past decades especially in rural population [16,54], and the time difference between the studies that could not consider the current level of fertility and mortality in other HDSSs elsewhere.

By substituting the national and regional infant and child death rates, the life expectancy at birth ranged from 48 years up to 55 years [16,42]. This is different from what we got from our study using the census data (Table 7; 62 years), and the current Ethiopian life expectancy of 66 years [26]. Since there is confirmed under-reporting of neonatal and children deaths in the community, we have a reason to believe that the life expectancy in Sidama is lower than the national estimates. Similarly, due to the possible omission of stillbirths and neonatal deaths, the number of births Sidama is smaller when compared to different scenarios of national and other HDSS studies [16,43].

As expected, women in the age group 25–29 years had a higher fertility rate when compared to younger age groups; as has been recorded elsewhere [16,51,55].

This study showed that there was no difference in fertility between highly educated women and uneducated women. This is different from the national health survey and study done in Butajira in 2009, in which uneducated women had more children when compared with women who completed secondary and above education [16,28]. The difference in this result might be the methodology used. The Butajira study used fertility as a count data to assess total children ever born to women in the reproductive age group and the national survey used data three years before the survey, whereas this study used births one year before the census. However, it could also be explained by the fact that uneducated women have an improvement in their contraceptive use, while educated women are having enough financial support to raise more children [52].

In the current study urban resident were more likely to give birth than their rural counterparts. This finding is contrary to other studies [16], in which urban residents had lower fertility except in Guatemala [56]. Moreover, the increasing rural- to- urban migration in Africa might contribute to higher fertility in urban areas [57]. This result might partially be explained also by disparity in utilizing birth registration between urban and rural communities [58,59].

The current study combined with previous studies suggests lower fertility, leading to demographic dividend. So the policymakers need to consider this transition and focus on interventions towards the increased working-age population. There is a significant gap in the community with age reporting and stillbirth and neonatal death reporting. This needs to be addressed by improving the vital registration systems in the community. Other demographic sites and subsequent rounds should consider possible measurement errors and try to lessen such limitations.

## Supplementary Material

Supplemental Material

## Data Availability

The data that support the findings of this study are openly available in Zenedo at https://zenodo.org/record/3888986#.X1-C3WgzbI, md5:32011f7c9ada1731854a964236e1beff [md5:79554c05f51dbe0177225b6794306cc3].

## Paper context

There is neither any demographic study done in Sidama recently, nor the quality of the data is assessed for its validity. The lower fertility rate is one sign of demographic transition that gives opportunity to utilize the work force created for development. However, the personal reporting of vital events has flaws that can be corrected by establishing a strong civil registration system supplemented by quality health and demographic surveillance data.
