# Supplementary material for: Births and deaths in Sidama in southern Ethiopia: findings from the 2018 Dale-Wonsho Health and Demographic Surveillance System (HDSS)
Source: Glob Health Action. 2020 Oct 29;13(1):1833511. doi: 10.1080/16549716.2020.1833511 (PMC7598947; doi:10.1080/16549716.2020.1833511)
Supplement: Supplemental Material [file ZGHA_A_1833511_SM7661.docx]

**Annex I: Supplemental materials**

**Supplementary material 1: Operational definitions**

**Household**: A person or group of related or unrelated persons who live together in the same dwelling unit(s), who acknowledge one adult male or female as the head of the household, who share the same housekeeping arrangements, and who are considered a single unit.

**Kebele**: The smallest administrative unit in the governmental structure [60].

**“got”:** Sub-division of kebele [61].

“**Luwa**”: Is cultural institution based on age grading system in which leaderships are changed every eight years [62].

**Under-age for educational status**: Based on the HDSS’s data collection format educational status is collected for those who are seven years of age and older. So under-age represents those below seven years of age [16].

**Under age for marital status**: Based on the HDSS’s data collection format marital status is collected for those who are ten years of age and older. So under age represents those below ten years of age.

**Under age for occupational status**: Based on the HDSS’s data collection format occupational status is collected for those who are ten years of age and older. So under age represents those below ten years of age.

**Basic education**: In the Ethiopian education system basic education refers to a formal education from grade 1-4 [63].

**Primary cycle**: In the Ethiopian education system basic primary cycle refers to a formal education from grade 5-8 [63].

**Secondary school**: In the Ethiopian education system secondary school education refers to a formal education from grade 9-10 [63].

**Preparatory school**: In the Ethiopian education system preparatory school education refers to a formal education from grade 11-12 [63].

**Higher education**: In the Ethiopian education system higher education refers to a formal education that ends up by awarding diploma or degree [63].

**Mortality**: Death of the children (neonatal, infant and under five years of age), adults and mothers occurring in the last one year before the survey [64].

**Birth outcomes**: All live births, still births or abortions encountered during the last 1 year before the survey [64].

**Skilled delivery**: A delivery care provided by skilled health professionals (doctors, midwives, health officer, nurses or health extension worker) during the last one year birth before the survey [16].

**Wealth index**Households are given scores based on the number and kinds of consumer goods they own; ownership of radio, television, refrigerator, telephone, bicycle, motorcycle, car, cattle, carriage animals, sheep (goats), chicken (poultry) and land were considered. In addition, housing characteristics was assessed by material of the roof and the floor, source of light, source of drinking water, toilet facilities and cooking material. All the variables are dichotomized and the scores are derived using principal component analysis and ranked in three quintiles [16].

Lowest quintile: those who score below the 3^rd^ quintile in wealth index ranking.

Middle quintile: those who score the middle 3^rd^ quintile in wealth index ranking.

Highest quintile: those who score the upper 3^rd^ quintile in wealth index ranking.

**Whipple’s index**: A measure of preference for ages ending in 0 and 5 [2].

Whipple’s index for ages ending in 5 =Population age 25+30+35…..+55 + 60 *100*5

Population age 23+ 24 +……..61 + 62

Whipple’s index for ages ending in 0 =Population age 30+40+ 50 +60 *100*5

Population age 23+ 24 +……..61 + 62

The range of values for Whipple’s index is <105 as highly accurate to >175 as very rough estimate on assessing reliability of age data [2].

**Myer’s blended index**: Is a more complex measure of age heaping. It considers preference (or avoidance) of age ending in each of the digits 0 to 9 in deriving overall age accuracy score [2].

The theoretical range of Myer’s Index is from 0 to 90, where 0 indicates no age heaping and 90 indicates the extreme case where all recorded ages end in the same digit.

**United Nations Joint Score:** Uses both age and sex ratio for computation up to the age of 70 by five year age group. Sex ratio is calculated by taking the difference between consecutive age groups taking the average of the absolute sum. Age ratio is calculated separately for male (ARM) and female (ARF) and the deviation from 100 is calculated. The mean of the absolute deviations of the two age ratios ARF and ARM are then summed to 3 times of the mean of the sex ratio differences (SR). UNJS= ARM+ARF + 3 (SR). UNJS between 0 and 19.9 is accurate, inaccurate if it is between 20 and 39.9, and highly in accurate if it is above 40 [40,41].

**Crude Birth Rate (CBR):** Births in a year divided by midyear population expressed per 1000 population [2].

**Total Fertility Rate (TFR**) is the number of children a woman would have if she survived to age 50 and throughout her reproductive life she experienced exactly the age specific fertility rates for the year in question [2].

**General fertility rate (GFR)**: Births during a year divided by midyear women population aged 15-49 expressed per 1000 women [2].

**Gross Reproductive Rate (GRR):** The average number of daughters a woman would have if she survived to at least age 50, and experienced the given female age specific fertility rate [2].

**Net Reproduction Rate (NRR)**: The average number of daughters a woman would have during her reproductive years given the fertility and mortality at the given rate [2].

**Crude Death Rate (CDR):** The number of deaths in a year divided by the total midyear population expresses per 1000 population [2].

**Infant Mortality Rate:** Number of deaths of under age of one year in a year divided by live births in the year expressed per 1000 live births [2].

**Child Mortality Rate**: The number of deaths of children between age one and five years old divided by the number of live births in a year expressed per 1000 live births [16].

**Under five morality rate**: The number of deaths of children <5 years old divided by the number of live births in a year expressed per 1000 live births [16].

**Crude rate of natural increase**: The difference between crude birth rate and crude death rate. It is a measure of current rate of population growth if net migration is not substantial [2].

**Median age**: Age that divides the population in two parts of equal size, that is, there are as many persons with ages above the median as there are with ages below the median [26].

**Dependency ratio**: Is the ratio of children and elderly population per working-age population expressed per 100 working population [2].

**Young dependency ratio**: Is the ratio of children population per working-age population expressed per 100 working population [2].

**Old dependency ratio**: Is the ratio of elderly population per working-age population expressed per 100 working population [2].

**Women of reproductive age group**: Women with in age group 15-49 years [16].

**Child /woman ratio**: The ratio of children aged 0-4 over women aged 15-49 [2].

**Sex ratio (birth):** The number of males births per 100 females births [2].

**Sex ratio (total)**: The number of males per 100 females [2].

**Average household size**: The average number of individuals in a household [16].

**Life expectancy at birth**: The average number of years of life expected by a hypothetical cohort of individuals who would be subject during all their lives to the mortality rates of a given period. It is expressed as years [26].

**How Life expectancy is calculated**: Abridged life table is used, where most of age groups are five years, it considers mortality data and some assumptions mentioned below [2].

| **Age interval (years)**  **(x,x+n)** | **_n_q _x_** | **_n_P_x_** | **_n_a_x_** | **l_x_** | **_n_d_x_** | **_n_L_x_** | **T_x_** | **e_x_** |
| --- | --- | --- | --- | --- | --- | --- | --- | --- |

**Where:**

**(x,x+n):** Interval or period of life between two exact ages stated in years**_._**

**_n_q_x :_** Probability of dying in the interval [x, x+n], given survival to age x.

**_n_P_x_**_:_ Probability of surviving in the interval [x, x+n], given survival to age x.

**_n_a_x_**: Average proportion of the time lived in the interval x to x+n by those who die during that interval.

**l_x:_** The number of persons alive at exact age x. l_0_ is an arbitrary number called the radix, usually set at 100,000.

**_n_d_x:_** The number of persons in the cohort who die in the age interval (x, x+ n).

**_n_L_x:_** Person-years lived between exact ages x and x+n.

**T_x:_** Total person-years of life contributed by the cohort after attaining age x.

**e_x:_** Average number of years a person aged x has to live.

**Supplementary material 2: Sampling**

Table 6: The study units of Dale and Wonsho districts, 2017/18, Sidama, Ethiopia.

| Kebele | Woreda | Rural / Urban | Random Sampling of population Proportional to size | Random sampling of HH Proportional to size | Sampled percentage |
| --- | --- | --- | --- | --- | --- |
| Danshe sire | Dale | Rural | 3,688 | 799 | 6.4 |
| Wayicho | Dale | Rural | 2,716 | 570 | 4.6 |
| Gidamo | Dale | Rural | 1,654 | 314 | 2.5 |
| Dagiya | Dale | Rural | 821 | 169 | 1.4 |
| Shoye | Dale | Rural | 3,085 | 625 | 5.0 |
| Hidakaliti | Dale | Rural | 1,970 | 381 | 3.0 |
| Magara | Dale | Rural | 2,661 | 576 | 4.6 |
| Aposto 01 | Dale | Urban | 2,972 | 646 | 5.2 |
| Mamana | Wonsho | Rural | 1,889 | 363 | 2.9 |
| Gishire | Wonsho | Rural | 1,355 | 258 | 2.1 |
| Bokaso rural | Wonsho | Rural | 978 | 198 | 1.6 |
| Bokaso 01 | Wonsho | Urban | 1,355 | 280 | 2.2 |
| **Total** | | | **25,144** | **5,179** | **41.4** |

**Supplementary material 3: Map of the study area**


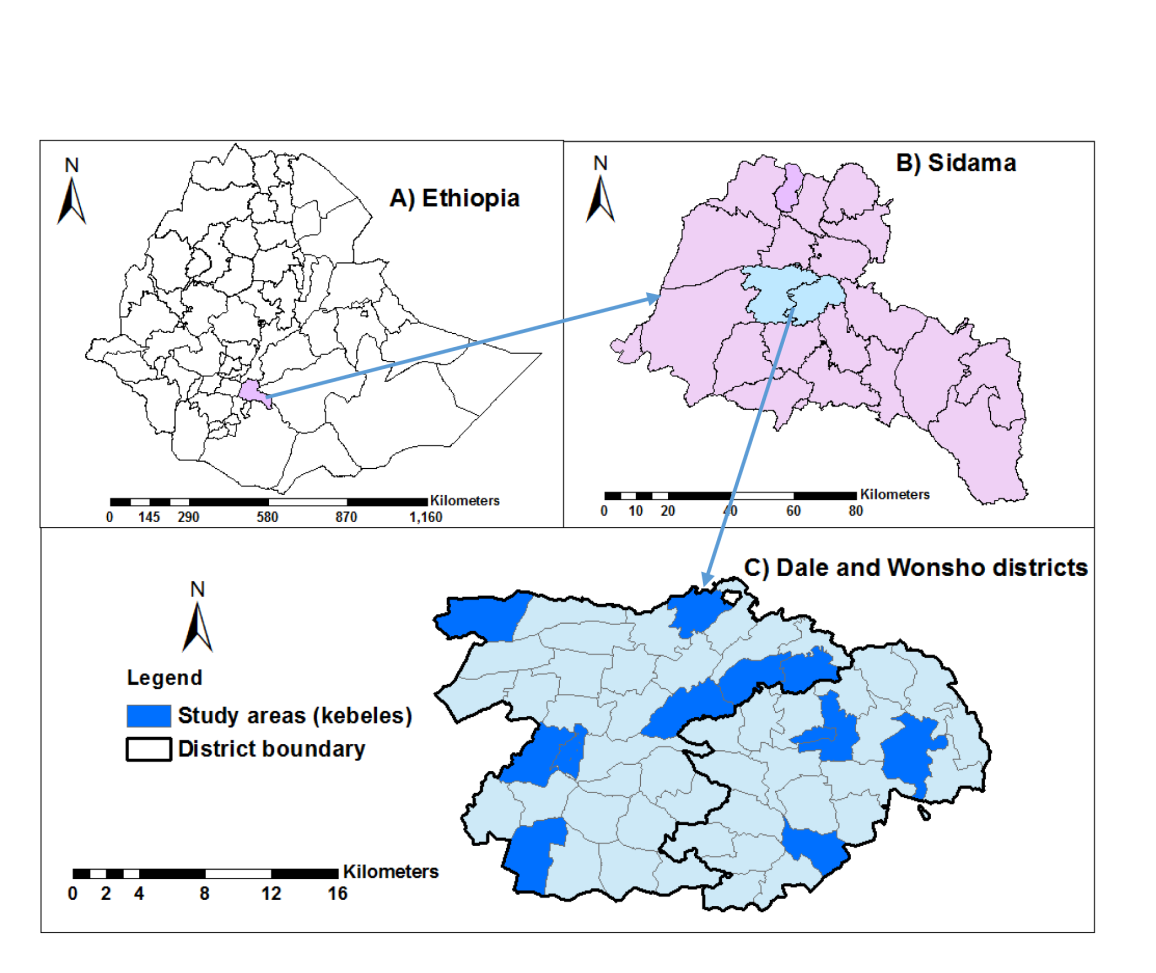


Figure 2: Map showing the study area

**Supplementary material 4: Background**

Table 7: Socio-economic and demographic characteristics of Dale and Wonsho districts’ population, 2017/18, Sidama, Ethiopia (N=25144).

| **S.N** | **Variable** | **Category** | **N** | **%** |
| --- | --- | --- | --- | --- |
|  | Sex | Male | 12,848 | 51.1 |
|  |  | Female | 12,296 | 48.9 |
|  | Age | 0-4 | 2,217 | 8.8 |
|  |  | 5-14 | 7,117 | 28.3 |
|  |  | 15-24 | 6,208 | 24.7 |
|  |  | 25-34 | 3,799 | 15.1 |
|  |  | 35-44 | 2,625 | 10.4 |
|  |  | 45-54 | 1,650 | 6.6 |
|  |  | 55-64 | 864 | 3.4 |
|  |  | 65-74 | 438 | 1.7 |
|  |  | 75-84 | 166 | 0.7 |
|  |  | >85 | 60 | 0.2 |
|  | Ethnicity | Sidama | 24,258 | 96.5 |
|  |  | Amhara | 438 | 1.7 |
|  |  | Gurage | 64 | 0.3 |
|  |  | Oromo | 150 | 0.6 |
|  |  | Wolayita | 141 | 0.6 |
|  |  | Others | 93 | 0.3 |
|  | Religion | Protestant | 22,322 | 88.8 |
|  |  | Muslim | 1,340 | 5.3 |
|  |  | Orthodox | 849 | 3.4 |
|  |  | Catholic | 517 | 2.0 |
|  |  | Others | 116 | 0.5 |

| **S.N** | **Variable** | **Category** | **N** | **%** |
| --- | --- | --- | --- | --- |
|  | Educational status | Illiterate | 4,842 | 19.3 |
|  |  | Read and Write | 710 | 2.8 |
|  |  | 1-4 | 7,596 | 30.2 |
|  |  | 5-6 | 3,434 | 13.7 |
|  |  | 7-12 | 4,677 | 18.6 |
|  |  | Higher level | 492 | 2.0 |
|  |  | Children <7 years old | 3,386 | 13.5 |
|  | Marital status | Married | 8,987 | 35.7 |
|  |  | Single | 10,063 | 40.0 |
|  |  | Divorced | 99 | 0.4 |
|  |  | Widowed | 602 | 2.4 |
|  |  | Children <10 years old | 5,393 | 21.5 |
|  | Occupation | Civil servant | 597 | 2.4 |
|  |  | Merchant | 1,531 | 6.1 |
|  |  | Driver | 56 | 0.2 |
|  |  | Farmer | 3,424 | 13.6 |
|  |  | Daily labourer | 1,125 | 4.5 |
|  |  | Housewife | 4,046 | 16.1 |
|  |  | Student (enrolled in school from primary to university level) | 8,088 | 32.2 |
|  |  | Housemaid | 128 | 0.5 |
|  |  | Others | 756 | 3.0 |
|  |  | Children <10 years old | 5,393 | 21.5 |
| 8. | Wealth Index | Lowest quintile | 8,398 | 33.4 |
|  |  | Middle quintile | 8,515 | 33.9 |
|  |  | Highest quintile | 8,231 | 32.7 |
| 9. | Residence | Rural | 20,817 | 82.8 |
|  |  | Urban | 4,327 | 17.2 |

Where, Illiterate: refers to those who cannot read and write and never at attended any form of education; Read and write: refers to those who did not attend formal school but can read and write.

**Supplementary material 5: Description of Birth**

| **Variables** | | **Male** | **Female** |
| --- | --- | --- | --- |
|  |  | **N (%)** | **N (%)** |
| Born in the last one year | | 305 (53.2) | 268 (46.8) |
| Type of birth | |  |  |
|  | Single | 299 (98.0) | 264 (98.5) |
|  | Twins | 4 (1.3) | 4 (1.5) |
|  | Multiple | 2 (0.7) | 0 (0.0) |
| Status of birth | |  |  |
|  | Live birth | 304 (99.7) | 268 (100.0) |
|  | Still birth | 1 (0.3) | 0 (0.0) |
| Place of birth | |  |  |
|  | Home | 121 (49.4) | 124 (50.6) |
|  | Health institution | 183 (56.3) | 142 (43.7) |
|  | Other places | 1 (33.3) | 2 (66.7) |
| Who attended the delivery | |  |  |
|  | Skilled Birth Attendants | 183 (60.0) | 143 (53.4) |
|  | Traditional Birth Attendants | 6 (2.0) | 16 (6.0) |
|  | Relatives | 20 (6.6) | 17 (6.3) |
|  | Neighbours | 93 (30.5) | 91 (34.0) |
|  | Others | 3 (1.0) | 1 (0.4) |

**Table 8:** Birth characteristics of Dale and Wonsho districts’ population, 2017/18, Sidama, Ethiopia (N=573).**Supplementary material 6: Causes of deaths**

Table 9: Causes of death in Dale and Wonsho districts’ population, 2017/18, Sidama, Ethiopia (N=132).

| **Causes of death** | **Male** | **Female** |
| --- | --- | --- |
|  | N (%) | N (%) |
| Unknown cause | 15 (50.0) | 15 (50.0) |
| Heart Failure | 9 (81.8) | 2 (18.2) |
| Aging | 5 (45.5) | 6 (54.6) |
| DM | 9 (90.0) | 1 (10.0) |
| Cancer + Liver cancer | 4 (40.0) | 6 (60.0) |
| Hypertension | 5 (62.5) | 3 (37.5) |
| TB | 3 (42.9) | 4 (57.1) |
| Sudden death & illness | 5 (71.4) | 2 (28.6) |
| Malaria | 2 (50.0) | 2 (50.0) |
| Gastro intestinal problem | 0 (0.0) | 7 (100.0) |
| Eye disease | 1 (50.0) | 1 (50.0) |
| Falling | 1 (50.0) | 1 (50.0) |
| Headache | 0 (0.0) | 2 (100.0) |
| Mental illness (suicide) | 1 (50.0) | 1 (50.0) |
| Renal disease | 1 (50.0) | 1 (50.0) |
| Battle fight | 1 (100.0) | 0 (0.0) |
| Car and Motor accident | 2 (100.0) | 0 (0.0) |
| Chocking | 0 (0.0) | 1 (100.0) |
| Drowning | 0 (0.0) | 1 (100.0) |
| Goiter | 0 (0.0) | 1 (100.0) |
| Hemorrhoid | 1 (100.0) | 0 (0.0) |
| Hunger and Malnutrition | 2 (100.0) | 0 (0.0) |
| Hypertension with Tumor | 1 (100.0) | 0 (0.0) |
| Liver disease | 1 (100.0) | 0 (0.0) |
| Liver disease with Malaria | 0 (0.0) | 1 (100.0) |
| Pneumonia + Lung damage | 2 (100.0) | 0 (0.0) |
| Nerve disease | 1 (100.0) | 0 (0.0) |
| Sharp object | 1 (100.0) | 0 (0.0) |
| Vomiting | 1 (100.0) | 0 (0.0) |

**Supplementary material 7: Life tables (Table 10-12)**

| **x** | **N** | **Px** | **Dx** | **nmx** | **Nax** | **nqx** | **npx** | **lx** | **ndx** | **nLx** | **Tx** | **e_x_** |
| --- | --- | --- | --- | --- | --- | --- | --- | --- | --- | --- | --- | --- |
| 0 | 1 | 641 | 7 | 0.01092 | 0.3 | 0.018565 | 0.981435 | 100000 | 1856.474 | 98700.47 | 6179769 | 61.79769 |
| 1 | 4 | 1576 | 3 | 0.001904 | 0.4 | 0.012183 | 0.987817 | 98143.53 | 1195.657 | 389704.5 | 6081068 | 61.96097 |
| 5 | 5 | 3175 | 1 | 0.000315 | 0.5 | 0.002362 | 0.997638 | 96947.87 | 229.0107 | 484166.8 | 5691364 | 58.7054 |
| 10 | 5 | 3943 | 3 | 0.000761 | 0.5 | 0.005706 | 0.994294 | 96718.86 | 551.9083 | 482214.5 | 5207197 | 53.83849 |
| 15 | 5 | 3685 | 2 | 0.000543 | 0.5 | 0.004071 | 0.995929 | 96166.95 | 391.453 | 479856.1 | 4724982 | 49.13312 |
| 20 | 5 | 2522 | 2 | 0.000793 | 0.5 | 0.005948 | 0.994052 | 95775.5 | 569.6401 | 477453.4 | 4245126 | 44.32372 |
| 25 | 5 | 2168 | 7 | 0.003229 | 0.5 | 0.024216 | 0.975784 | 95205.86 | 2305.492 | 470265.6 | 3767673 | 39.57396 |
| 30 | 5 | 1631 | 0 | 0 | 0.5 | 0 | 1 | 92900.36 | 0 | 464501.8 | 3297407 | 35.49402 |
| 35 | 5 | 1479 | 5 | 0.003381 | 0.5 | 0.025355 | 0.974645 | 92900.36 | 2355.486 | 458613.1 | 2832905 | 30.49402 |
| 40 | 5 | 1146 | 8 | 0.006981 | 0.5 | 0.052356 | 0.947644 | 90544.88 | 4740.57 | 440873 | 2374292 | 26.22227 |
| 45 | 5 | 772 | 7 | 0.009067 | 0.5 | 0.068005 | 0.931995 | 85804.31 | 5835.138 | 414433.7 | 1933419 | 22.53289 |
| 50 | 5 | 878 | 9 | 0.010251 | 0.5 | 0.076879 | 0.923121 | 79969.17 | 6147.972 | 384475.9 | 1518986 | 18.99464 |
| 55 | 5 | 336 | 6 | 0.017857 | 0.5 | 0.133929 | 0.866071 | 73821.2 | 9886.768 | 344389.1 | 1134510 | 15.36835 |
| 60 | 5 | 528 | 10 | 0.018939 | 0.5 | 0.142045 | 0.857955 | 63934.43 | 9081.595 | 296968.2 | 790120.7 | 12.3583 |
| 65 | 5 | 241 | 8 | 0.033195 | 0.5 | 0.248963 | 0.751037 | 54852.84 | 13656.31 | 240123.4 | 493152.5 | 8.990465 |
| 70 | 5 | 197 | 11 | 0.055838 | 0.5 | 0.418782 | 0.581218 | 41196.53 | 17252.35 | 162851.8 | 253029.1 | 6.142 |
| 75 | 5 | 83 | 8 | 0.096386 | 0.5 | 0.722892 | 0.277108 | 23944.18 | 17309.04 | 76448.27 | 90177.33 | 3.766149 |
| 80 | 5 | 83 | 12 | 0.144578 | 0.5 | 1.084337 | -0.08434 | 6635.133 | 7194.722 | 15188.86 | 13729.06 | 2.069146 |
| 85+ |  | 60 | 23 | 0.383333 |  | 1 | 0 | -559.59 | -559.59 | -1459.8 | -1459.8 | 2.608696 |

Table 10: Life expectancy for both sexes in Dale and Wonsho districts’ population, 2017/18, Sidama, Ethiopia (N=25144).

Table 11: Life expectancy for males in Dale and Wonsho districts’ population, 2017/18, Sidama, Ethiopia (N=12,848).

| **x** | **n** | **Px** | **Dx** | **nmx** | **nax** | **nqx** | **npx** | **lx** | **ndx** | **nLx** | **Tx** | **e_x_** |
| --- | --- | --- | --- | --- | --- | --- | --- | --- | --- | --- | --- | --- |
| 0 | 1 | 335 | 3 | 0.008955 | 0.3 | 0.015224 | 0.984776 | 100000 | 1522.388 | 98934.33 | 6321721 | 63.21721 |
| 1 | 4 | 803 | 1 | 0.001245 | 0.4 | 0.00797 | 0.99203 | 98477.61 | 784.8776 | 392026.7 | 6222786 | 63.18986 |
| 5 | 5 | 1581 | 1 | 0.000633 | 0.5 | 0.004744 | 0.995256 | 97692.73 | 463.438 | 487305.1 | 5830760 | 59.68468 |
| 10 | 5 | 2005 | 2 | 0.000998 | 0.5 | 0.007481 | 0.992519 | 97229.3 | 727.4012 | 484328 | 5343455 | 54.95725 |
| 15 | 5 | 1928 | 2 | 0.001037 | 0.5 | 0.00778 | 0.99222 | 96501.9 | 750.7928 | 480632.5 | 4859127 | 50.35266 |
| 20 | 5 | 1208 | 0 | 0 | 0.5 | 0 | 1 | 95751.1 | 0 | 478755.5 | 4378494 | 45.72787 |
| 25 | 5 | 1008 | 4 | 0.003968 | 0.5 | 0.029762 | 0.970238 | 95751.1 | 2849.735 | 471631.2 | 3899739 | 40.72787 |
| 30 | 5 | 880 | 0 | 0 | 0.5 | 0 | 1 | 92901.37 | 0 | 464506.8 | 3428108 | 36.90051 |
| 35 | 5 | 731 | 2 | 0.002736 | 0.5 | 0.02052 | 0.97948 | 92901.37 | 1906.321 | 459741 | 2963601 | 31.90051 |
| 40 | 5 | 614 | 4 | 0.006515 | 0.5 | 0.04886 | 0.95114 | 90995.05 | 4446.012 | 443860.2 | 2503860 | 27.51644 |
| 45 | 5 | 406 | 2 | 0.004926 | 0.5 | 0.036946 | 0.963054 | 86549.03 | 3197.624 | 424751.1 | 2059999 | 23.80153 |
| 50 | 5 | 393 | 5 | 0.012723 | 0.5 | 0.09542 | 0.90458 | 83351.41 | 7953.379 | 396873.6 | 1635248 | 19.61872 |
| 55 | 5 | 185 | 3 | 0.016216 | 0.5 | 0.121622 | 0.878378 | 75398.03 | 9170.031 | 354065.1 | 1238375 | 16.4245 |
| 60 | 5 | 312 | 7 | 0.022436 | 0.5 | 0.168269 | 0.831731 | 66228 | 11144.13 | 303279.7 | 884309.6 | 13.3525 |
| 65 | 5 | 158 | 6 | 0.037975 | 0.5 | 0.28481 | 0.71519 | 55083.87 | 15688.44 | 236198.2 | 581030 | 10.5481 |
| 70 | 5 | 131 | 3 | 0.022901 | 0.5 | 0.171756 | 0.828244 | 39395.42 | 6766.389 | 180061.1 | 344831.8 | 8.753092 |
| 75 | 5 | 64 | 5 | 0.078125 | 0.5 | 0.585938 | 0.414063 | 32629.03 | 19118.57 | 115348.7 | 164770.6 | 5.049816 |
| 80 | 5 | 58 | 6 | 0.103448 | 0.5 | 0.775862 | 0.224138 | 13510.46 | 10482.25 | 41346.66 | 49421.88 | 3.658046 |
| 85+ |  | 48 | 18 | 0.375 |  | 1 | 0 | 3028.206 | 3028.206 | 8075.217 | 8075.217 | 2.666667 |

Table 12: Life expectancy for females in Dale and Wonsho districts’ population, 2017/18, Sidama, Ethiopia (N=12,296)

| **x** | **n** | **Px** | **Dx** | **nmx** | **nax** | **nqx** | **npx** | **lx** | **ndx** | **nLx** | **Tx** | **e_x_** |
| --- | --- | --- | --- | --- | --- | --- | --- | --- | --- | --- | --- | --- |
| 0 | 1 | 306 | 4 | 0.013072 | 0.3 | 0.022222 | 0.977778 | 100000 | 2222.222 | 98444.44 | 6001394 | 60.01394 |
| 1 | 4 | 773 | 2 | 0.002587 | 0.4 | 0.016559 | 0.983441 | 97777.78 | 1619.089 | 387225.3 | 5902950 | 60.37108 |
| 5 | 5 | 1594 | 0 | 0 | 0.5 | 0 | 1 | 96158.69 | 0 | 480793.4 | 5515725 | 57.36065 |
| 10 | 5 | 1938 | 1 | 0.000516 | 0.5 | 0.00387 | 0.99613 | 96158.69 | 372.1311 | 479863.1 | 5034931 | 52.36065 |
| 15 | 5 | 1757 | 0 | 0 | 0.5 | 0 | 1 | 95786.56 | 0 | 478932.8 | 4555068 | 47.55436 |
| 20 | 5 | 1314 | 2 | 0.001522 | 0.5 | 0.011416 | 0.988584 | 95786.56 | 1093.454 | 476199.2 | 4076135 | 42.55436 |
| 25 | 5 | 1160 | 3 | 0.002586 | 0.5 | 0.019397 | 0.980603 | 94693.1 | 1836.72 | 468873.7 | 3599936 | 38.01688 |
| 30 | 5 | 751 | 0 | 0 | 0.5 | 0 | 1 | 92856.38 | 0 | 464281.9 | 3131062 | 33.71941 |
| 35 | 5 | 748 | 3 | 0.004011 | 0.5 | 0.03008 | 0.96992 | 92856.38 | 2793.14 | 457299.1 | 2666780 | 28.71941 |
| 40 | 5 | 532 | 4 | 0.007519 | 0.5 | 0.056391 | 0.943609 | 90063.24 | 5078.754 | 437619.3 | 2209481 | 24.53255 |
| 45 | 5 | 366 | 5 | 0.013661 | 0.5 | 0.102459 | 0.897541 | 84984.49 | 8707.427 | 403153.9 | 1771862 | 20.84924 |
| 50 | 5 | 485 | 4 | 0.008247 | 0.5 | 0.061856 | 0.938144 | 76277.06 | 4718.169 | 369589.9 | 1368708 | 17.9439 |
| 55 | 5 | 151 | 3 | 0.019868 | 0.5 | 0.149007 | 0.850993 | 71558.89 | 10662.75 | 331137.6 | 999118.3 | 13.96218 |
| 60 | 5 | 216 | 3 | 0.013889 | 0.5 | 0.104167 | 0.895833 | 60896.14 | 6343.348 | 288622.4 | 667980.7 | 10.96918 |
| 65 | 5 | 83 | 2 | 0.024096 | 0.5 | 0.180723 | 0.819277 | 54552.8 | 9858.939 | 248116.6 | 379358.3 | 6.953967 |
| 70 | 5 | 66 | 8 | 0.121212 | 0.5 | 0.909091 | 0.090909 | 44693.86 | 40630.78 | 121892.3 | 131241.7 | 2.936459 |
| 75 | 5 | 19 | 3 | 0.157895 | 0.5 | 1.184211 | -0.18421 | 4063.078 | 4811.54 | 8286.541 | 9349.356 | 2.301053 |
| 80 | 5 | 25 | 6 | 0.24 | 0.5 | 1.8 | -0.8 | -748.462 | -1347.23 | -374.231 | 1062.816 | -1.42 |
| 85+ |  | 12 | 5 | 0.416667 |  | 1 | 0 | 598.7694 | 598.7694 | 1437.047 | 1437.047 | 2.4 |

Where; X=exact age, n= interval between two exact ages stated in years; Px=Population in x, x+n age group; Dx= number of deaths in x, x+n age group; nmx=age specific mortality rate in x, x+n age group; _n_a_x_=average proportion of the time lived in the interval x to x+n by those who die during that interval _; n_q _x=_Probability of dying in the interval [x, x+n], given survival to age x; _n_P_x_ =Probability of surviving in the interval [x, x+n], given survival to age x; l_x=_The number of persons alive at exact age x, ; _n_d_x=_The number of persons in the cohort who die in the age interval (x, x+ n);_n_L_x=_Person-years lived between exact ages x and x+n; T_x=_Total person-years of life contributed by the cohort after attaining age x; e_x=_Average Number of years a person aged x has to live.

**Supplementary material 8: ASFR and FASFR**

Table 13: Estimates of annual ASFRs and FASFRs for all women 15-49 years in Dale and Wonsho districts, 2017/18, Sidama, Ethiopia.

| **Age-group** | **Births** | **Number of women** | **Rate/woman** | **Rate/1000 women** |
| --- | --- | --- | --- | --- |
| ASFR |  |  |  |  |
| 15-19 | 44 | 1757 | 0.025 | 25 |
| 20-24 | 167 | 1314 | 0.127 | 127 |
| 25-29 | 166 | 1160 | 0.143 | 143 |
| 30-34 | 97 | 751 | 0.129 | 129 |
| 35-39 | 72 | 748 | 0.095 | 95 |
| 40-44 | 24 | 532 | 0.045 | 45 |
| 45-49 | 3 | 366 | 0.008 | 8 |
| Female ASFR |  |  |  |  |
| 15-19 | 22 | 1757 | 0.013 | 13 |
| 20-24 | 82 | 1314 | 0.062 | 62 |
| 25-29 | 74 | 1160 | 0.064 | 64 |
| 30-34 | 43 | 751 | 0.057 | 57 |
| 35-39 | 35 | 748 | 0.047 | 47 |
| 40-44 | 11 | 532 | 0.021 | 21 |
| 45-49 | 1 | 366 | 0.003 | 3 |

**Supplementary material 9: GRR and NRR**

Table 14: Estimates of annual GRR and NRR for all women 15-49 years in Dale and Wonsho districts, 2017/18, Sidama, Ethiopia

| **Age group** | **No of Women of reproductive age group** | **Male birth** | **Female birth** | **total birth** | **Female ASFR** | **Mid-point of age group** | **FASFR*Mid-point of age group** | **Prob. surviving(lx)** | **Lx (stationary popn)** | **exp. female birth** |
| --- | --- | --- | --- | --- | --- | --- | --- | --- | --- | --- |
| 15-19 | 1757 | 22 | 22 | 44 | 0.012521 | 17.5 | 0.219123506 | 0.979489266 | 4.89744633 | 0.061322606 |
| 20-24 | 1314 | 85 | 82 | 167 | 0.062405 | 22.5 | 1.404109589 | 0.979489266 | 4.882882156 | 0.304715629 |
| 25-29 | 1160 | 92 | 74 | 166 | 0.063793 | 27.5 | 1.754310345 | 0.973663596 | 4.843055723 | 0.308953555 |
| 30-34 | 751 | 54 | 43 | 97 | 0.057257 | 32.5 | 1.860852197 | 0.963558693 | 4.817793464 | 0.275852355 |
| 35-39 | 748 | 37 | 35 | 72 | 0.046791 | 37.5 | 1.754679144 | 0.963558693 | 4.781146962 | 0.22371677 |
| 40-44 | 532 | 13 | 11 | 24 | 0.020677 | 42.5 | 0.878759398 | 0.948900092 | 4.682399669 | 0.096816535 |
| 45-49 | 366 | 2 | 1 | 3 | 0.002732 | 47.5 | 0.129781421 | 0.924059775 | 2.310149439 | 0.006311884 |
|  |  |  |  |  | 0.266177 |  | 8.001615601 |  |  | 1.277689334 |

GRR= 5*sum (ASFR)= 5*0.26617=1.33

NRR= GRR*Probability of surviving to the mean of age specific fertility distribution (30.06 years in our case) 1.33*0.964 = 1.28 or Sum of (exp female birth)
